# Supplementary material for: Active learning-guided optimization of cell-free biosensors for lead testing in drinking water
Source: Nat Commun. 2025 Dec 20;17:261. doi: 10.1038/s41467-025-66964-6 (PMC12783771; doi:10.1038/s41467-025-66964-6)
Supplement: Supplementary file 1 — Supplementary Information [file 41467_2025_66964_MOESM1_ESM.pdf]

Supplementary Information for

Active learning-guided optimization of cell-free biosensors for lead testing in drinking water

Brenda M. Wang[1*], Nicole Chiang[2*], Holly M. Ekas[3,4,5], Dylan M. Brown[3,5], Garrett Dildine[6], Tyler J. Lucci[3,5], Siyuan Feng[5,7], Vanessa Bly[5,8], Jean-François Gaillard[6], Julius B. Lucks[3,5,9,10], Ashty S. Karim[3,4,5], Diwakar Shukla[2,11,12,13], and Michael C. Jewett[1,2,3,4,5,#]

Affiliations

[1]Department of Bioengineering, Stanford University, Stanford, CA 94305, USA
[2]Department of Chemical and Biomolecular Engineering, University of Illinois at Urbana-Champaign, Urbana, IL, 61801, USA
[3]Department of Chemical and Biological Engineering, Northwestern University, Evanston, IL 60208, USA
[4]Chemistry of Life Processes Institute, Northwestern University, Evanston, IL 60208, USA
[5]Center for Synthetic Biology, Northwestern University, Evanston, IL 60208, USA
[6]Department of Civil and Environmental Engineering, Northwestern University, Evanston, IL 60208, USA
[7]Department of Biomedical Engineering, Northwestern University, Evanston, IL 60208, USA
[8]Bridges/Puentes: Justice Collective of the Southeast, Chicago, IL, USA
[9]Center for Water Research, Northwestern University, Evanston, IL 60208, USA
[10]Interdisciplinary Biological Sciences Graduate Program, Northwestern University, Evanston, IL 60208, USA
[11]Department of Bioengineering, University of Illinois at Urbana-Champaign, Urbana, IL, 61801, USA
[12]Department of Chemistry, University of Illinois at Urbana-Champaign, Urbana, IL, 61801, USA
[13]Beckman Institute for Advanced Science and Technology, University of Illinois at Urbana-Champaign, Urbana, IL, 61801, USA
[*]These authors contributed equally: Brenda M. Wang and Nicole Chiang.

[#]To whom correspondence should be addressed:

Michael Jewett, Stanford University, 443 Via Ortega, Stanford, CA 94305,
mjewett@stanford.edu; Tel (+1) 650 497 0112

Supplementary Figures

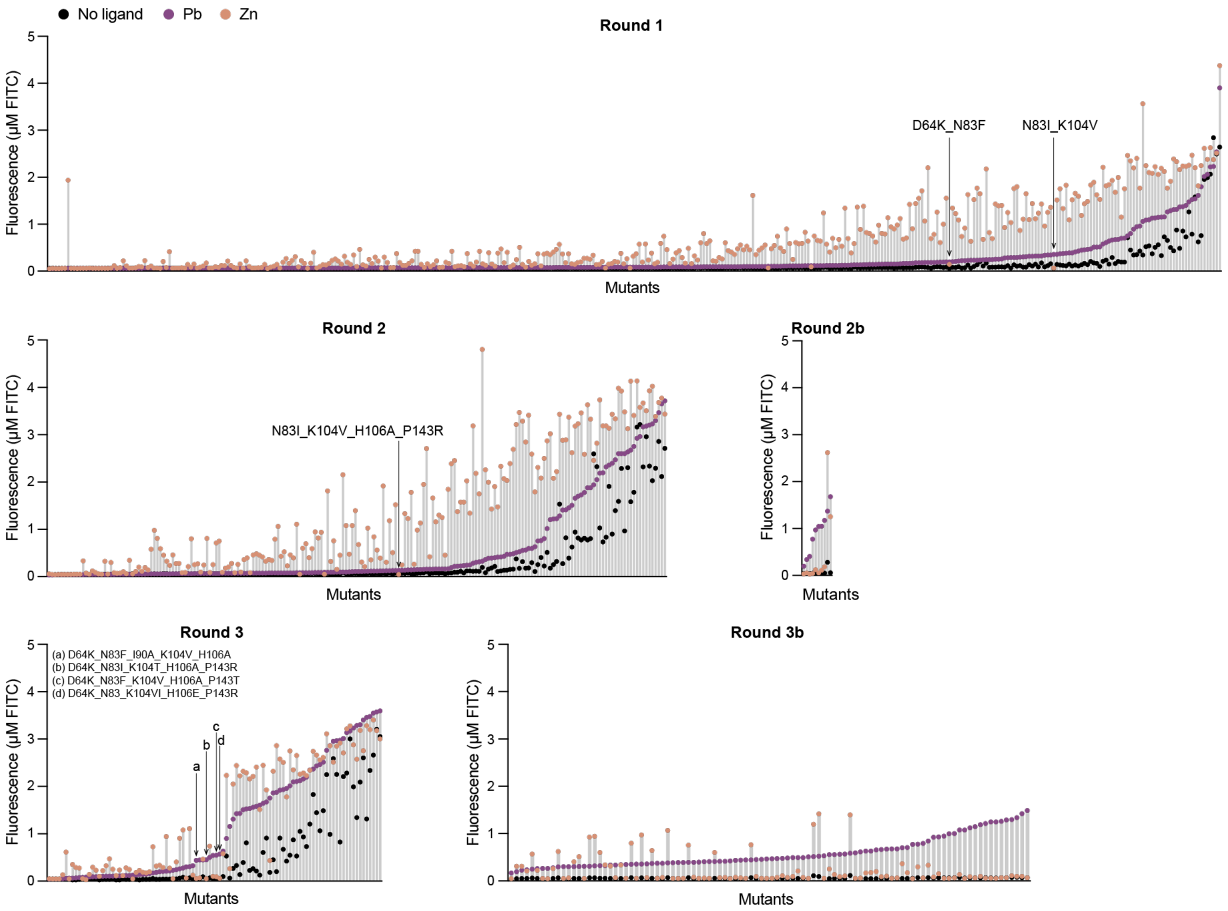

Supplementary Figure 1 | Fluorescent activity of mutants from high-throughput screen. For each round, a library of mutants was screened against a low concentration of lead (1 µM), high concentration of zinc (30 µM), and no ligand condition. Reactions were set up by the Echo acoustic liquid handling robot into black 384-well PCR plates at 1 µL. Mutants in Round 1, 2, and 4 that are used to inform rational library design in Rounds 2b and 3b are highlighted on the graphs. Data represent the average of three biological replicates (n = 3). Source data are provided as a Source Data file.

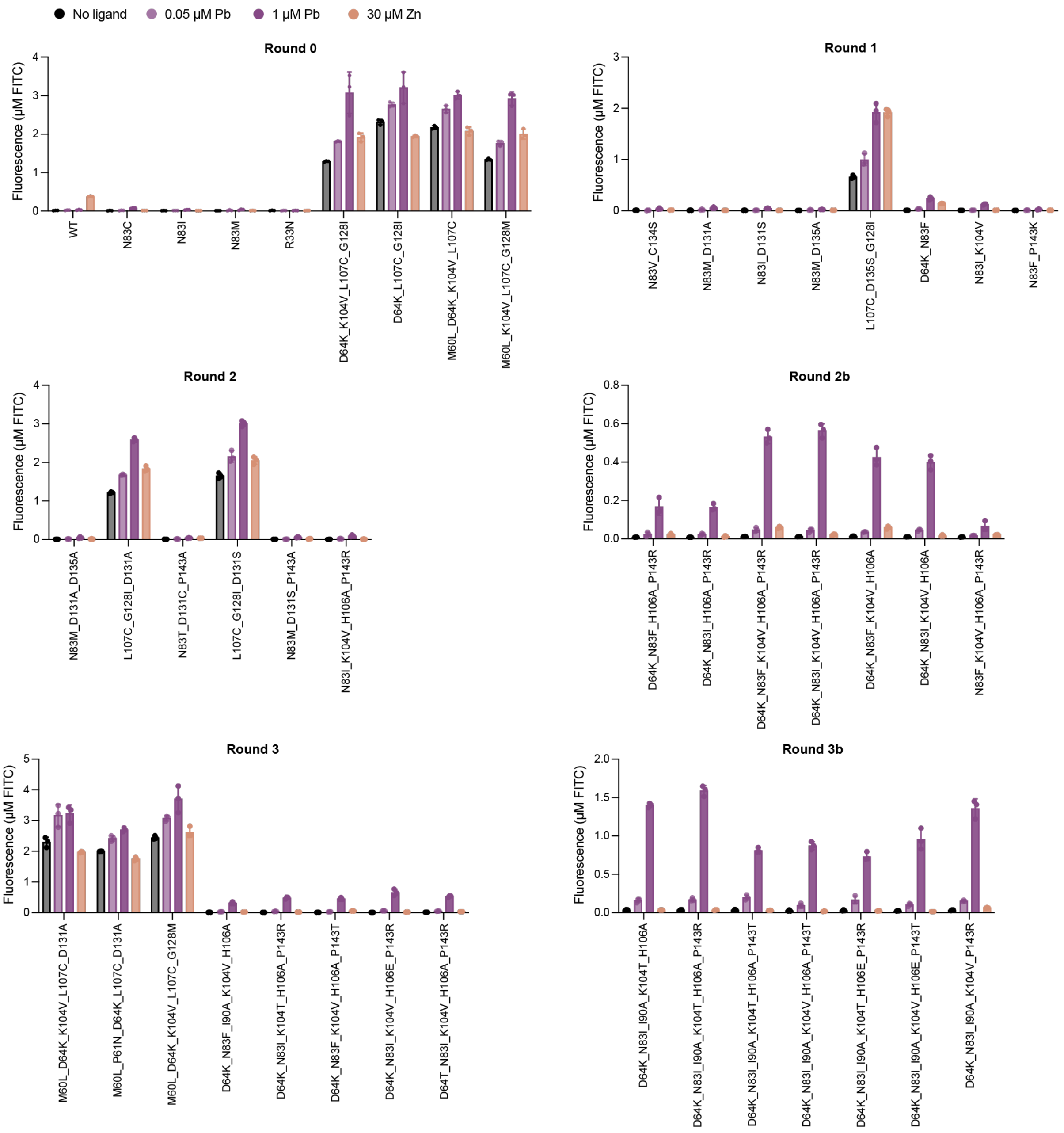

Supplementary Figure 2 | Validating the top mutants from high-throughput screen. For each round, 10 mutants were identified for manual validation (i.e., hand pipetting). The top 5 mutants with the highest normalized fold change to lead and low normalized fold change to zinc (i.e., normalized fold change to zinc < 1) were selected for manual validation. Similarly, 5 mutants were selected based on normalized dynamic range values. Duplicates from these two rankings were removed. Mutants were validated by manually setting up 10 µL reactions. Data are presented as mean values +/- SD of three biological replicates (n = 3). Source data are provided as a Source Data file.

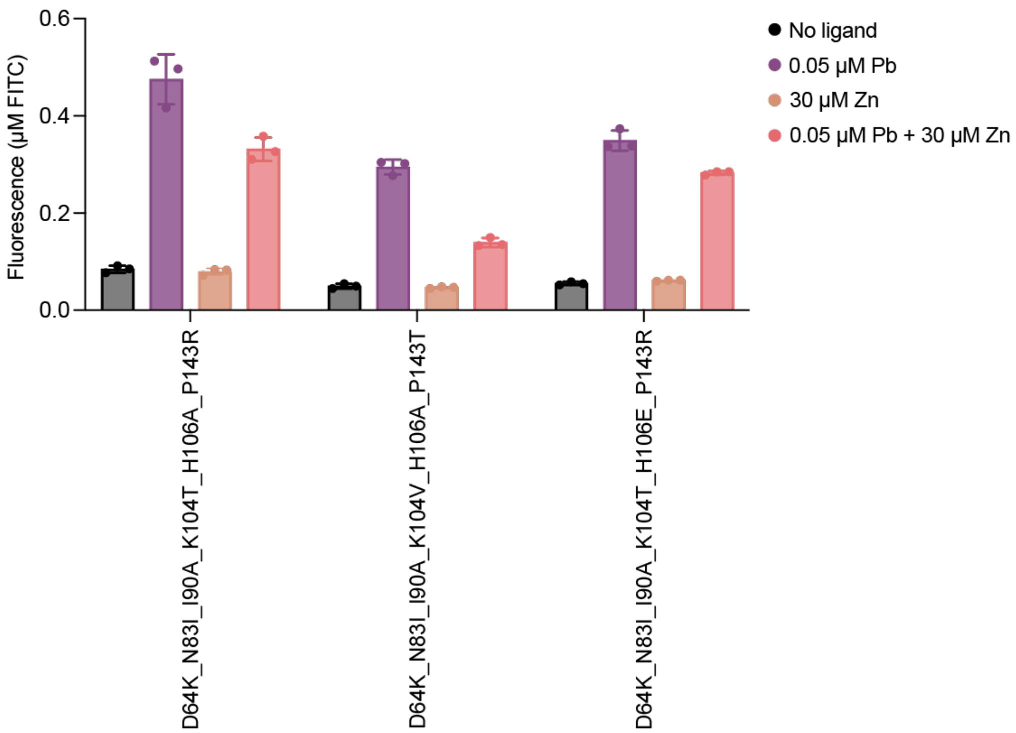

Supplementary Figure 3 | CFE biosensing reactions using PbrR enriched extract. Extract enriched in a PbrR mutant were created for the top 3 mutants identified in Round 3b. Enriched extracts are at 0.1% v/v of the reaction. Each mutant was tested against four conditions: (1) no ligand, (2) lead near the EPA action level of 48 nM (0.05 µM), (2) 30 µM zinc, and (4) 0.05 µM lead and 30 µM zinc. Data are presented as mean values +/- SD of three biological replicates (n = 3). Source data are provided as a Source Data file.

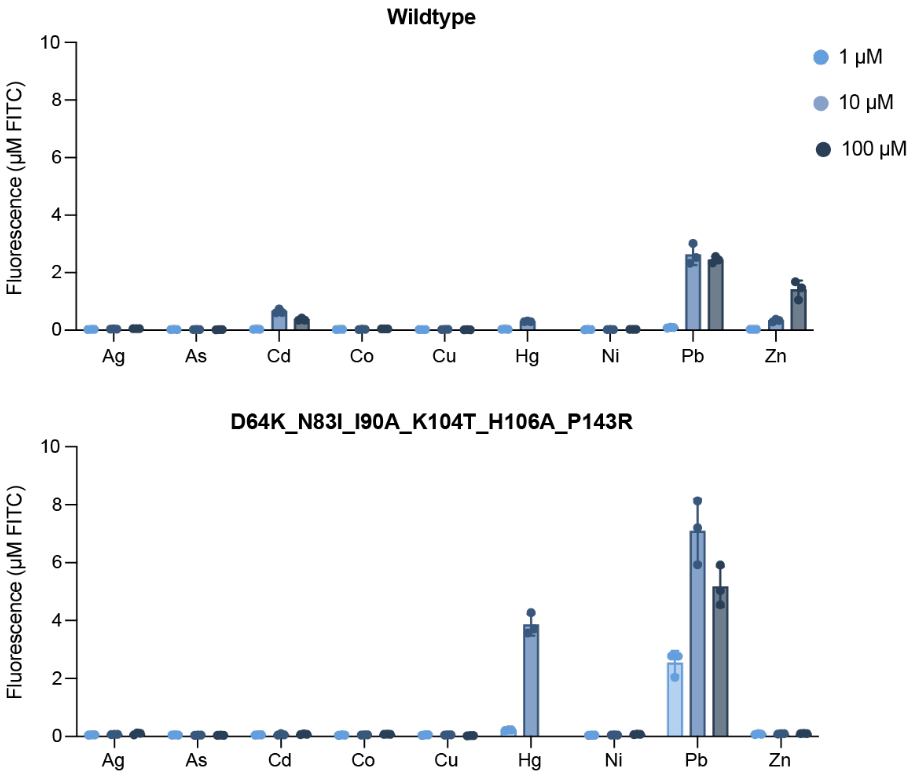

Supplementary Figure 4 | Testing the sensitivity of the best PbrR mutant to other metal ligands. The cross-reactivities of wildtype PbrR and mutant D64K_N83I_I90A_K104T_H106A_P143R to similar divalent metal ions were characterized at three different concentrations (1 µM, 10 µM, and 100 µM). These biosensing reactions used extracts enriched in PbrR. PbrR was not tested against 100 µM mercury due to toxicity issues at that concentration. Data are presented as mean values +/- SD of three biological replicates (n = 3). Source data are provided as a Source Data file.

Question 1

A B C D E

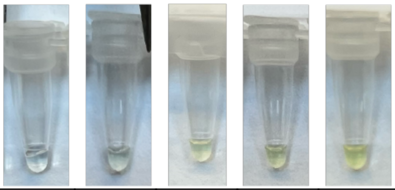

Responses	0	9	12	0	0
Abs_{385nm}	0.26 ± 0.00	0.45 ± 0.02	0.58 ± 0.03	0.69 ± 0.05	0.90 ± 0.09

Question 2

A B C D E

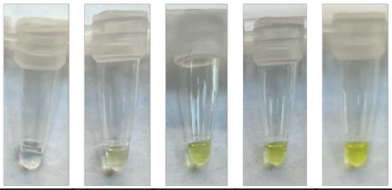

Responses	0	21	0	0	0
Abs_{385nm}	0.25 ± 0.03	0.86 ± 0.06	1.68 ± 0.05	2.37 ± 0.02	3.00 ± 0.09

Supplementary Figure 5 | Determining a 'visible by eye' absorbance value for the catechol reporter. In a poll, we asked 21 people to select the tube in which they observe an initial color change from clear (first tube) to yellow in each picture. Based on the poll responses, we set the "visible by eye" line in Figure 6G at Abs_{385nm} = 0.75. This is approximately the average between the absorbance values of Tube C in Question 1 and Tube B in Question 2. Absorbance values represent the mean +/- SD of three biological replicates (*n* = 3). Source data are provided as a Source Data file.

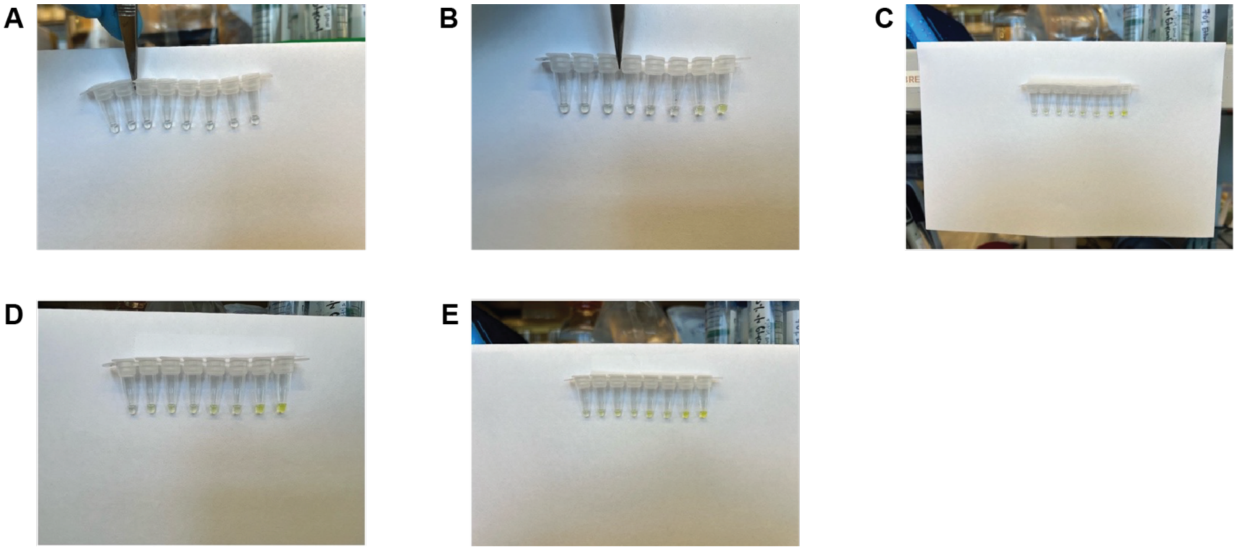

Supplementary Figure 6 | Uncropped photos used in Supplementary Figure 5. Freeze-dried biosensor reactions were rehydrated with 15-μL municipal water samples containing varying concentrations of lead and incubated at 30°C for 12 hours. Pictures of the reactions were taken at (A) 44 min, (B) 264 min, (C) 320 min, (D) 360 min, and (E) 430 min. From each picture, tubes #3 and #7 (counting from left to right) were used in Question 1 and Question 2, respectively, of the poll in Supplementary Figure 5.

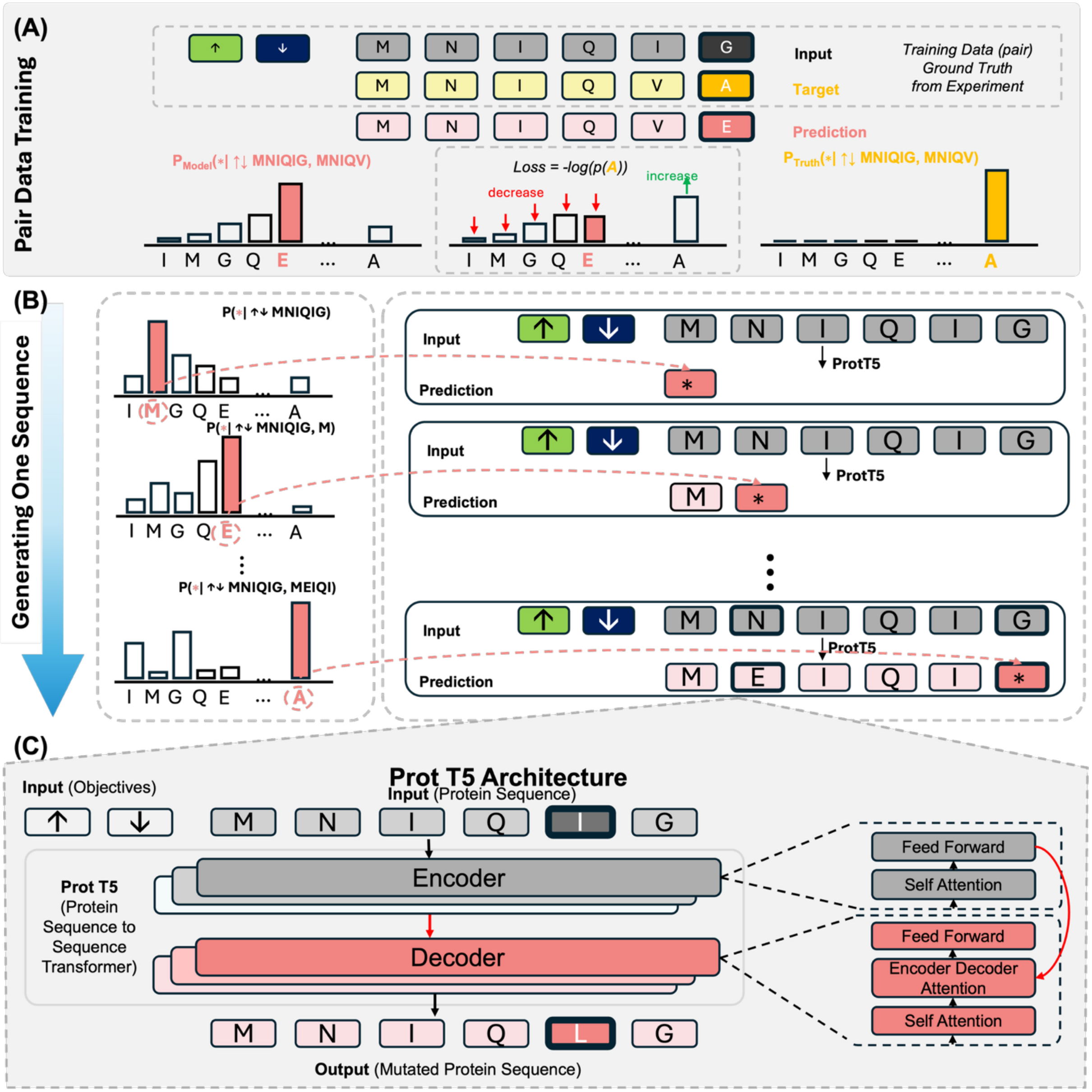

Supplementary Figure 7 | Overview of the model architecture and workflow. (A) Training procedure where the transformer-based sequence-to-sequence model receives directional tokens and input sequences to autoregressively predict target sequences. (B) Inference procedure generating novel protein sequences conditioned on user-defined multi-objective direction tokens and seed sequences. (C) Detailed model architecture showing the transformer encoder-decoder components and added special tokens for directionality.

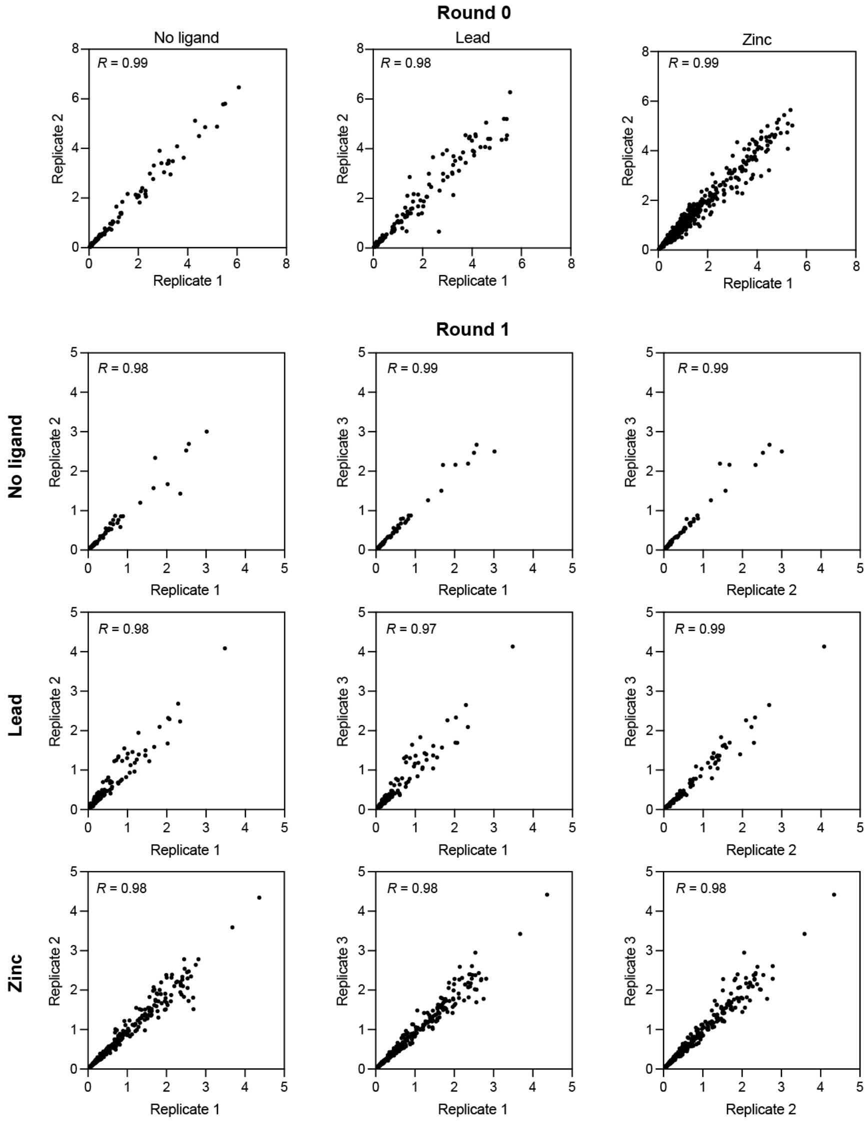

Supplementary Figure 8 | Analysis of the variability in screening assay replicates. Data collected from the screening assay in Figures 3 and 4 in the main text. Representative parity plots of the two biological replicates from Round 0 and three biological replicates from Round 1. The consistency of replicates is shown by the fit to the line y = x and corresponding *R* value displayed on the chart. Source data are provided as a Source Data file.

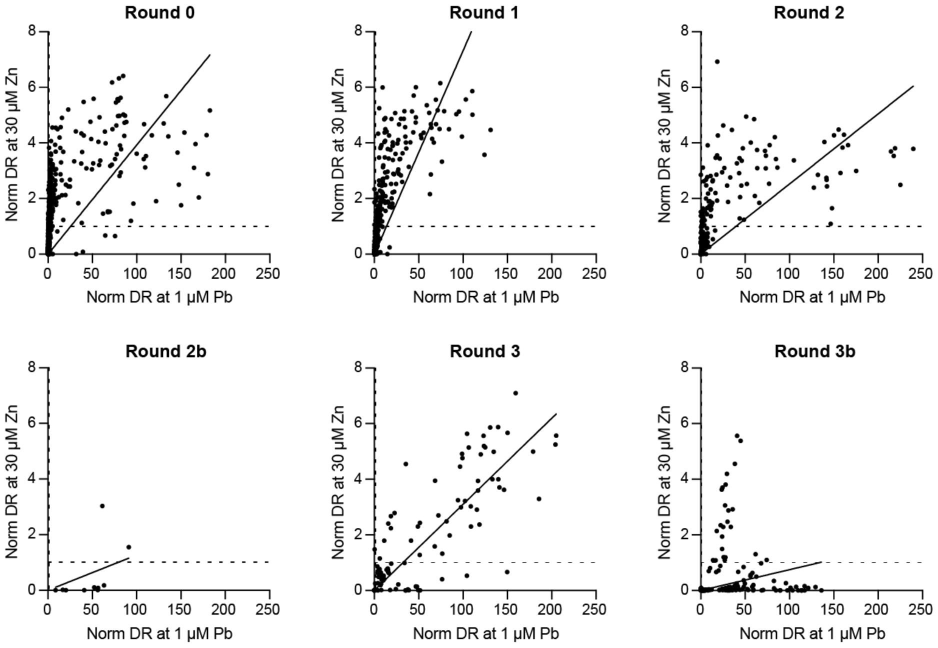

Supplementary Figure 9 | Normalized dynamic range. Normalized dynamic range (DR) was calculated from the data collected in each round and used to train the model beginning in Round 2. Mutant data in the screening assay were collected in biological triplicate (n = 3) and the average normalized DR for each mutant is used in the scatterplots. Line of best fit is plotted on each graph. Source data are provided as a Source Data file.

Supplementary Note 1

Require: Experimental sequences $\mathcal{X} = \{X^{(1)}, \ldots, X^{(M)}\}$, multi-objective function values $f(X) = [f_1(X), \ldots, f_j(X)]$, noise thresholds $\{\tau_\kappa\}_{\kappa=1}^{j}$, pre-trained model P_θ

Ensure: Fine-tuned model P_θ

Initialize training set $\mathcal{D} \leftarrow \emptyset$
for all pairs $(X_1, X_2) \in \mathcal{X} \times \mathcal{X}$, $X_1 \neq X_2$ **do**
  Initialize label vector $\mathbf{d} \leftarrow []$
  for $\kappa = 1$ **to** j **do**
    $\Delta f_\kappa \leftarrow f_\kappa(X_2) - f_\kappa(X_1)$
    if $\Delta f_\kappa > \tau_\kappa$ **then**
      Append `<inc>` to $\mathbf{d}$
    else
      Append `<dec>` to $\mathbf{d}$
    end if
  end for
  Add training example $(\mathbf{d}, X_1, X_2)$ to $\mathcal{D}$
end for
Balance $\mathcal{D}$ over all observed label combinations in {`<inc>`, `<dec>`}^{j}
Train model P_θ by maximizing

$$\mathcal{L}(\theta) = \sum_{(\mathbf{d}, X_1, X_2) \in \mathcal{D}} \log P_\theta(X_2 \mid \mathbf{d}, X_1)$$

using token-level cross-entropy loss and parameter-efficient finetuning (LoRA)

Supplementary Note 1 | Pseudocode for training the multi-objective controlled extrapolation model.

Supplementary Note 2

Require: Fine-tuned model P_θ, seed sequences $\mathcal{S}$, direction vector $\mathbf{d} \in \{\texttt{<inc>}, \texttt{<dec>}\}^j$, sampling parameter k

Ensure: Designed sequences $\mathcal{Y}$

Initialize output set $\mathcal{Y} \leftarrow \emptyset$

for all seed $X \in \mathcal{S}$ **do**

  Construct prompt: $\texttt{prompt} \leftarrow [\mathbf{d}, X]$

  Sample candidates $\{Y^{(1)}, \ldots, Y^{(k)}\} \sim P_\theta(\cdot \mid \texttt{prompt})$ via top-k sampling

  Filter candidates by mutation constraints (e.g., edit distance from wild-type)

  Add filtered candidates to $\mathcal{Y}$

end for

return $\mathcal{Y}$

Supplementary Note 2 | Pseudocode for sequence generation using the trained model with directional conditioning.

Supplementary Tables

Parameter	Value	Description
Foundation model	Rostlab/prot_t5_xl_uniref50	Pretrained ProtT5 XL UniRef50 model
Special tokens added	<inc>, <dec>	Added as special tokens for directionality
Max input sequence length	150	Max length for source and target sequences
Tokenizer	T5Tokenizer	From HuggingFace, with added special tokens
LoRA rank (r)	16	Low-rank dimension for LoRA fine-tuning
LoRA alpha	32	LoRA scaling factor
LoRA dropout	0.05	Dropout in LoRA layers
Optimizer	AdamW	With weight decay
Learning rate	1.00E-04	Higher than full fine-tuning
Batch size	1	Per device
Number of epochs	1	Single epoch fine-tuning
Generation max tokens	150	Maximum tokens generated per sequence
Sampling strategy	Top-k sampling	top_k=10, stochastic sampling
Temperature (generation)	0.7 ~ 1.0	Controls randomness of output sequences
Number of sequences returned	20	Number of sequences generated For each seed sequence
Early stopping	Enabled	Stops generation once EOS token is produced
Device used	CUDA GPU or CPU	Depending on availability

Supplementary Table 1 | Model architecture and training parameters used for fine-tuning the protein sequence generation model. The base model is ProtT5, a transformer encoder-decoder model. Parameter-efficient fine-tuning was performed using Low-Rank Adaptation (LoRA), targeting the query (q) and value (v) projection layers in the decoder self-attention blocks. Training was conducted using the Hugging Face Seq2SeqTrainer.
