# Supplementary material for: Active learning-guided optimization of cell-free biosensors for lead testing in drinking water
Source: Nat Commun. 2025 Dec 20;17:261. doi: 10.1038/s41467-025-66964-6 (PMC12783771; doi:10.1038/s41467-025-66964-6)
Supplement: Supplementary file 3 — Description of Additional Supplementary Files [file 41467_2025_66964_MOESM3_ESM.pdf]

Description of Additional Supplementary Files

File Name: Supplementary Data 1
Description: This file contains the nucleotide sequences for all PbrR mutants screened in this study.
