# Supplementary material for: Active learning-guided optimization of cell-free biosensors for lead testing in drinking water
Source: Nat Commun. 2025 Dec 20;17:261. doi: 10.1038/s41467-025-66964-6 (PMC12783771; doi:10.1038/s41467-025-66964-6)
Supplement: Supplementary file 5 — Reporting Summary [file 41467_2025_66964_MOESM5_ESM.pdf]

nature portfolio

Corresponding author(s): Michael Jewett

Last updated by author(s): Oct 17, 2025

Reporting Summary

Nature Portfolio wishes to improve the reproducibility of the work that we publish. This form provides structure for consistency and transparency in reporting. For further information on Nature Portfolio policies, see our Editorial Policies and the Editorial Policy Checklist.

Statistics

For all statistical analyses, confirm that the following items are present in the figure legend, table legend, main text, or Methods section.

n/a	Confirmed	
☐	☒	The exact sample size (*n*) for each experimental group/condition, given as a discrete number and unit of measurement
☐	☒	A statement on whether measurements were taken from distinct samples or whether the same sample was measured repeatedly
☒	☐	The statistical test(s) used AND whether they are one- or two-sided<br>*Only common tests should be described solely by name; describe more complex techniques in the Methods section.*
☒	☐	A description of all covariates tested
☒	☐	A description of any assumptions or corrections, such as tests of normality and adjustment for multiple comparisons
☐	☒	A full description of the statistical parameters including central tendency (e.g. means) or other basic estimates (e.g. regression coefficient) AND variation (e.g. standard deviation) or associated estimates of uncertainty (e.g. confidence intervals)
☒	☐	For null hypothesis testing, the test statistic (e.g. *F*, *t*, *r*) with confidence intervals, effect sizes, degrees of freedom and *P* value noted<br>*Give P values as exact values whenever suitable.*
☒	☐	For Bayesian analysis, information on the choice of priors and Markov chain Monte Carlo settings
☒	☐	For hierarchical and complex designs, identification of the appropriate level for tests and full reporting of outcomes
☒	☐	Estimates of effect sizes (e.g. Cohen's *d*, Pearson's *r*), indicating how they were calculated

Our web collection on statistics for biologists contains articles on many of the points above.

Software and code

Policy information about availability of computer code

Data collection	All data were collected using stated instruments and associated commercially available software. Commercial software used includes: Gen5 Version 2.09.2 (BioTek Synergy Neo2 or H1) for measuring GFP fluorescence or absorbance and Qtegra ISDS (ThermoFisher iCAP-Q) for measuring municipal water metal content.
Data analysis	Data analysis and figure generation were conducted using Excel Version 16.19.1, ChimeraX Version 1.9, Prism Version 10.4.2, Jupiter Notebook Version 7.2.2, Chai-1, and Python 3.12. Custom scripts are available on GitHub (https://github.com/ShuklaGroup/multiobjective_controlled_extrapolation).

For manuscripts utilizing custom algorithms or software that are central to the research but not yet described in published literature, software must be made available to editors and reviewers. We strongly encourage code deposition in a community repository (e.g. GitHub). See the Nature Portfolio guidelines for submitting code & software for further information.

Data

Policy information about availability of data

All manuscripts must include a data availability statement. This statement should provide the following information, where applicable:
- Accession codes, unique identifiers, or web links for publicly available datasets
- A description of any restrictions on data availability
- For clinical datasets or third party data, please ensure that the statement adheres to our policy

All data presented in this manuscript are available in the Source Data file. DNA sequences for the PbrR mutants used in this study are included in the Source Data file. The Uniprot accession code for wildtype PbrR is Q58AJ5 [https://www.uniprot.org/uniprotkb/Q58AJ5/entry]. The Addgene accession code for the wildtype PbrR plasmid is 167215 [https://www.addgene.org/167215/]. The Addgene accession codes for the sfGFP and catechol reporter plasmids are 167222 [https://www.addgene.org/167222/] and 167254 [https://www.addgene.org/167254/], respectively.

Research involving human participants, their data, or biological material

Policy information about studies with human participants or human data. See also policy information about sex, gender (identity/presentation), and sexual orientation and race, ethnicity and racism.

Reporting on sex and gender	N/A
Reporting on race, ethnicity, or other socially relevant groupings	N/A
Population characteristics	N/A
Recruitment	N/A
Ethics oversight	N/A

Note that full information on the approval of the study protocol must also be provided in the manuscript.

Field-specific reporting

Please select the one below that is the best fit for your research. If you are not sure, read the appropriate sections before making your selection.

☒ Life sciences ☐ Behavioural & social sciences ☐ Ecological, evolutionary & environmental sciences

For a reference copy of the document with all sections, see nature.com/documents/nr-reporting-summary-flat.pdf

Life sciences study design

All studies must disclose on these points even when the disclosure is negative.

Sample size	Heatmaps in Figure 3 are n = 2 biological replicates. All other data are n = 3 biological replicates. Sample sizes were determined based on precedent and were not calculated.
Data exclusions	No data was excluded.
Replication	All attempts at replication were successful and are stated throughout the manuscript text and figure legends. Biological replicates were composed of individual CFE reactions. For initial workflow prototyping, replication experiments were set-up on separate days using separate sets of reagents and found to be consistent.
Randomization	No randomization was performed.
Blinding	No blinding was performed as all experiments required experimenter knowledge for proper set-up. Al data analysis was done based on objective data measurements that could not be directly influenced by the researcher (e.g. plate reader values).

Reporting for specific materials, systems and methods

We require information from authors about some types of materials, experimental systems and methods used in many studies. Here, indicate whether each material, system or method listed is relevant to your study. If you are not sure if a list item applies to your research, read the appropriate section before selecting a response.

Materials & experimental systems

n/a	Involved in the study
☒	☐ Antibodies
☒	☐ Eukaryotic cell lines
☒	☐ Palaeontology and archaeology
☒	☐ Animals and other organisms
☒	☐ Clinical data
☒	☐ Dual use research of concern
☒	☐ Plants

Methods

n/a	Involved in the study
☒	☐ ChIP-seq
☒	☐ Flow cytometry
☒	☐ MRI-based neuroimaging

Plants

Seed stocks: N/A

Novel plant genotypes: N/A

Authentication: N/A
